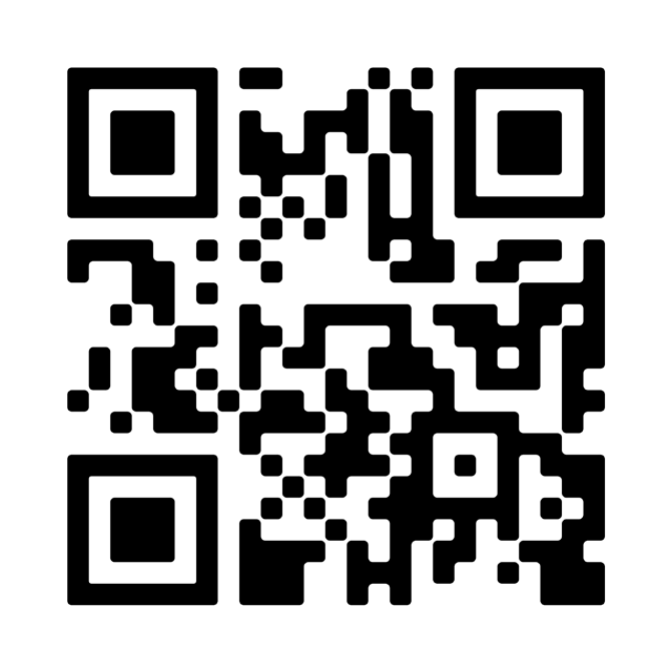# Robotic-assisted excision of diffuse adenomyosis

**DOI:** 10.52054/FVVO.16.3.034

**Published:** 2024-09-30

**Authors:** Y Youssef, I Alkatout, JM Ayoubi, A Feki, G Moawad

**Affiliations:** Division of Minimally invasive Gynecology, Department of Obstetrics and Gynecology, Maimonides Medical Center, Brooklyn, NY, USA, 11219; Kiel School of Gynecological Surgery, Department of Gynecology and Obstetrics, University Hospitals Schleswig-Holstein, Campus Kiel, Kiel, Germany , 21405; Department of Obstetrics and Gynecology and Reproductive Medicine, Hopital Foch–Faculté de Médecine Paris, 92150 Suresnes, France; Department of Obstetrics and Gynecology, HFR—Hòpital Fribourgeois, Chemin des Pensionnats 2-6, 1708 Fribourg, Switzerland; Department of Obstetrics and Gynecology, The George Washington University Hospital, Washington, DC 20037, USA; The Center for Endometriosis and Advanced Pelvic Surgery, Chevy Chase, MD, USA, 20815

**Keywords:** Adenomyosis, robotic surgery, uterus-sparing surgery

## Abstract

**Background:**

Adenomyosis is a chronic, debilitating condition characterised by the presence of endometrial- like glands and stroma within the myometrium. While hysterectomy remains the definitive treatment, uterus- sparing surgeries may be a possible option for patients desiring to maintain fertility. Surgical management, along with medical treatment and/or Assisted Reproductive Technology (ART), can improve outcomes.

**Objectives:**

To provide a step-by-step video demonstration of robotic-assisted excision of diffuse adenomyosis affecting the posterior uterine wall.

**Materials and Methods:**

This video article describes the use of a robotic platform in conjunction with intracavitary indocyanine green (ICG) for the uterus-sparing excision of diffuse adenomyosis.

**Main outcome measures:**

Perioperative data, specifics of the surgical approach, and both objective and subjective outcomes of this surgical approach.

**Results:**

A 38-year-old nulligravid patient with a history of chronic pelvic pain and infertility underwent surgical management of adenomyosis following two unsuccessful IVF cycles. The excisional surgery resulted in minimal blood loss (60 ml) and the patient was discharged on the same day of surgery with no complications.

**Conclusion:**

In select patients, robotic-assisted surgical management of diffuse adenomyosis can be advantageous. Leveraging the benefits of robotic technology, combined with appropriate surgical techniques, facilitates the performance of extensive surgeries with minimal morbidity and favourable outcomes.

## Learning objective

In this surgical tutorial, we illustrate techniques aimed at reducing blood loss during uterus-sparing procedures. Strategic surgical planning, guided by preoperative imaging, is vital to determine the most suitable technique. The surgery’s objective is to remove all evident pathology while ensuring the preservation of myometrial integrity. Utilising the robotic 3D camera and Firefly technology enables simultaneous use of intracavitary indocyanine green (ICG), optimising the excision of the diseased tissue. Moreover, the precision and dexterity of the robotic arms support meticulous suturing of the myometrial defect, which is pivotal in improving surgical outcomes.

## Introduction

Adenomyosis is strongly associated with infertility and can significantly impact the outcomes of infertility treatments. It has been identified as the primary cause of infertility in over 80% of patients and in more than 30% of patients who have experienced previous ART failures (Puente et al., 2016). Additionally, adenomyosis is correlated with various adverse pregnancy outcomes, including uterine rupture, abnormal placentation, preterm labour, premature rupture of membranes, and foetal growth restriction. Improved pregnancy and live birth rates, along with lower miscarriage rates, have been reported following the surgical excision of the disease (Hashimoto et al., 2018). The combination of surgery with medical treatment and/or ART has also been explored as potentially beneficial, although the data has been inconsistent due to heterogeneity (Moawad et al., 2024). Various surgical techniques, ranging from excisional to flap techniques, have been reported, depending on the extent of the disease and the number of uterine walls involved. These techniques can be performed either through an open or a minimally invasive approach using conventional laparoscopy or robotic-assisted methods. However, due to the absence of high-quality evidence, there is no consensus on the optimal surgical approach.

## Patients and methods

A 38-year-old nulligravid patient with a history of chronic pelvic pain, dysmenorrhea, and infertility for 2 years was referred to our centre for surgical intervention. The patient had previously undergone two unsuccessful IVF cycles. Her preoperative AMH was 1.4 ng/ml. During her first IVF cycle, five embryos were produced; one embryo (grade 3AA) was transferred, and the remaining embryos were frozen. In the second attempt, one frozen embryo (grade 4AB) was transferred after endometrial preparation as per protocol. Unfortunately, both cycles were unsuccessful, resulting in implantation failure.

**Figure 1:**
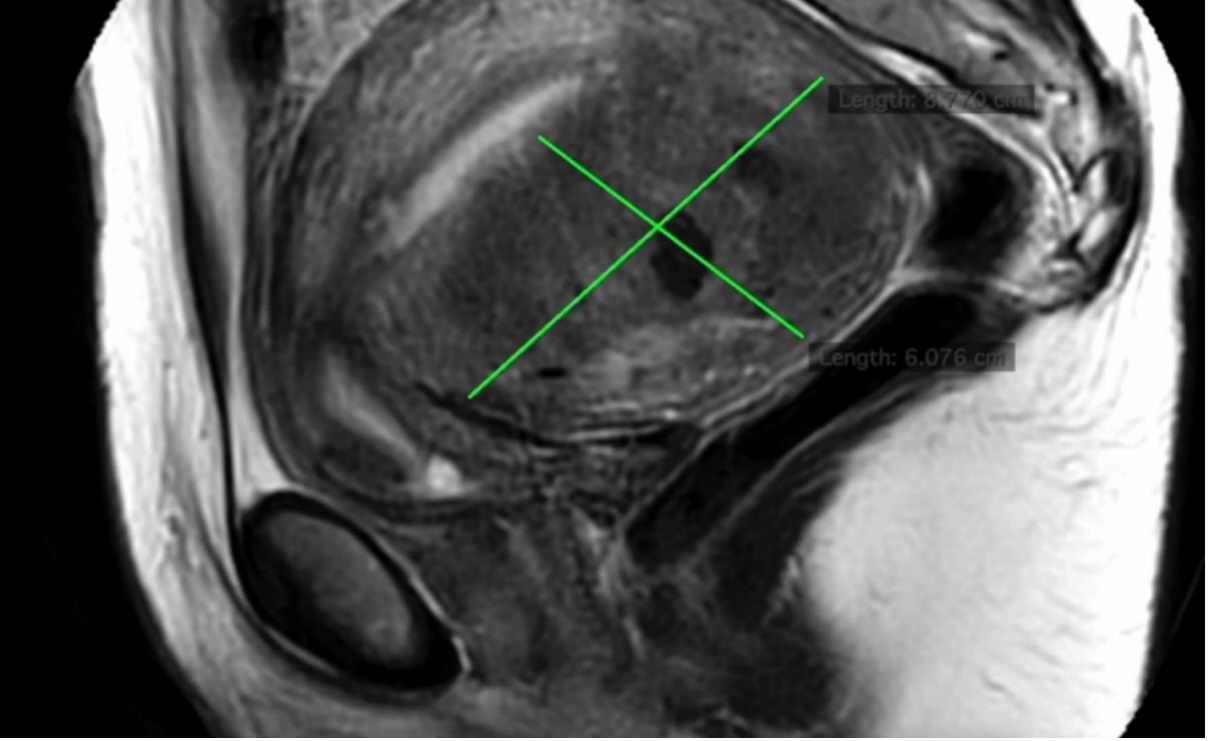
MRI image showing a posterior wall diffuse adenomyosis (8.7 x 6.0 cm) with a thickened junctional zone.

Following thorough counselling on the risks, benefits, and alternatives, including repeating another IVF cycle, receiving a pre-cycle GnRH agonist, or opting for surgical management, she chose to undergo a uterus-sparing excisional surgery utilising a minimally invasive approach. A preoperative MRI scan revealed diffuse adenomyosis on the posterior wall of the uterus, measuring 8.7 x 6.0 cm, with a thickened junctional zone (> 12 mm) (Figure 1).

## Results

**Figure 2:**
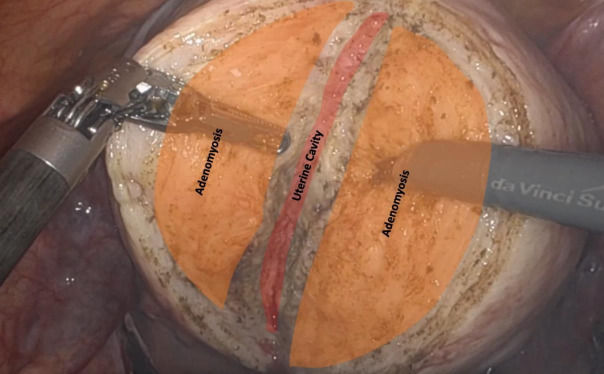
A vertical incision along the posterior wall of the uterus, carried down to bisect the adenomyotic tissue and facilitate its excision in pieces.

**Figure 3:**
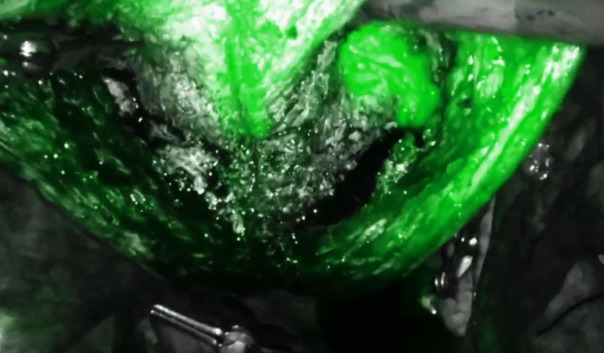
The diluted intracavitary indocyanine green guidance allows for preserving 0.5 cm of myometrium around the cavity and detects any cavitary breaches.

Robotic-assisted laparoscopic excision of diffuse adenomyosis was performed using the following steps:

1. Techniques to minimise blood loss were employed, including temporary occlusion of the major blood supply. This was achieved by applying bulldog vascular clamps on the uterine vessels at their origin and on the utero-ovarian vessels at the utero-ovarian ligaments bilaterally.
2. Subserosal injection of 20 ml of diluted vasopressin (10 IU diluted in 100 ml of normal saline) was administered.
3. A vertical incision was made along the posterior uterine wall. Adenomyotic tissue was excised in pieces, maintaining a layer of myometrium (0.5 cm) subserosally (Figure 2).
4. Intracavitary diluted ICG (1 ml in 100 ml of saline) was used to assist with complete excision of the diseased tissue while preserving a layer of myometrium (0.5 cm) around the uterine cavity and to detect any breaches into the endometrial cavity (Figure 3).
5. Excess serosa was excised. The myometrium was approximated by suturing each ipsilateral subserosal flap to the ipsilateral central myometrium, followed by a single layer to approximate the serosa.
6. Intracavitary diluted ICG was utilised to check for tubal patency at the end of the procedure, and haemostasis was ensured.

The surgery was uncomplicated with a blood loss of 60 ml and the patient was discharged on the same day. She was advised to postpone future attempts to conceive until 6 months after surgery. At her 6-week postoperative visit, she was evaluated and will obtain imaging before her next IVF cycle as planned.

## Discussion

Uterus-sparing surgeries have been described in the literature using both open and minimally invasive approaches. Various techniques have been proposed to excise and reconstruct the uterine walls in adenomyosis surgery, including the classic excision technique, transverse H technique, wedge resection, overlapping flaps, and triple flap techniques. Comparative studies between different approaches or surgical techniques are lacking, leaving the choice to the discretion of the surgeons based on their experience (Grimbizis et al., 2014). This video article demonstrates a step-by-step approach to a minimally invasive excisional technique in a case of diffuse posterior adenomyosis. The procedure was performed using the Da Vinci Xi robotic platform.

In this approach, we highlight the versatility of the robotic platform, which enables the surgeon to perform complex surgeries with minimal morbidity. The primary goal of the surgery is to achieve complete excision of the diseased tissue while maintaining adequate residual myometrium beneath the serosa and around the cavity. A 3D vision of the surgical field, along with the use of diluted intracavitary ICG, helps in identifying challenging surgical planes, ensuring complete excision, and detecting any breach into the cavity. Furthermore, the 7-degree freedom of motion of the robotic arms facilitates precise suturing of the myometrial bed, minimising the risk of hematoma formation and subsequent morbidity. Preoperative imaging plays a crucial role in surgical mapping to overcome the haptic feedback limitations of the robotic platform.

Adenomyosis has a detrimental effect on fertility and pregnancy outcomes. In patients undergoing Assisted Reproductive Technology (ART), the likelihood of getting pregnant is reduced by 31%- 43%, and the live birth rate (LBR) decreases by 55%, with a significant increase in the miscarriage rate (Odds Ratio [OR] 2.1 to 3.4) (Vercellini et al., 2023). The negative impact of adenomyosis also continues to affect pregnancy outcomes, with risks including an elevated likelihood of experiencing preeclampsia (OR 4.35 to 7.87), preterm delivery (OR 2.65–3.09), delivering an infant small-for- gestational-age (SGA) (OR 2.86 to 3.90), and postpartum haemorrhage (OR 2.90) (Vercellini et al., 2023). Previous meta-analyses have shown favourable results after uterus-sparing surgeries, with a range of pregnancy rates (38.5-49.1%) depending on the extent of the disease (Tan et al., 2018). Postoperative GnRH agonist therapy has been shown to improve outcomes, however, results have been inconsistent (Tan et al., 2018). ART in conjunction with surgery showed improved outcomes in one meta-analysis, while it showed comparable outcomes to natural conception after surgery in another (Tan et al., 2018; Jiang et al., 2023).

## Conclusions

In conclusion, uterus-sparing excisional surgery could be a treatment consideration for patients with symptomatic adenomyosis who failed medical therapy, and in cases of infertility lasting several years or repeated failure of assisted reproductive technology. A minimally invasive approach utilising the robotic platform is feasible and reproducible, albeit with proper preoperative surgical planning and adequate surgical techniques.

## Video scan (read QR)

https://vimeo.com/944074262/207dcdc418?share=copy

**Figure:**